# The loss of Tm7sf gene accelerates skin papilloma formation in mice

**DOI:** 10.1038/srep09471

**Published:** 2015-03-25

**Authors:** I. Bellezza, L. Gatticchi, R. del Sordo, M. J. Peirce, A. Sidoni, R. Roberti, A. Minelli

**Affiliations:** 1 Dipartimento di Medicina Sperimentale, Università di Perugia, Polo Unico Sant'Andrea delle Fratte, p.le Gambuli, Perugia, 06132; Italia

## Abstract

The 3β-hydroxysterol Δ14-reductase, encoded by the *Tm7sf2* gene, is an enzyme involved in cholesterol biosynthesis. Cholesterol and its derivatives control epidermal barrier integrity and are protective against environmental insults. To determine the role of the gene in skin cholesterol homeostasis, we applied 12-o-tetradecanoylphorbol-13-acetate (TPA) to the skin of *Tm7sf2*^+/+^ and *Tm7sf2*^-/-^ mice. TPA increased skin cholesterol levels by inducing de novo synthesis and up-take only in *Tm7sf2*^+/+^ mouse, confirming that the gene maintains cholesterol homeostasis under stress conditions. Cholesterol sulfate, one of the major players in skin permeability, was doubled by TPA treatment in the skin of wild-type animals but this response was lost in *Tm7sf2*^-/-^ mice. The expression of markers of epidermal differentiation concomitant with farnesoid-X-receptor and p38 MAPK activation were also disrupted in *Tm7sf2*^-/-^ mice. We then subjected *Tm7sf2*^+/+^ and *Tm7sf2*^-/-^ mice to a classical two-stage skin carcinogenesis protocol. We found that the loss of *Tm7sf2* increased incidence and multiplicity of skin papillomas. Interestingly, the null genotype showed reduced expression of nur77, a gene associated with resistance to neoplastic transformation. In conclusion, the loss of *Tm7sf2* alters the expression of proteins involved in epidermal differentiation by reducing the levels of cholesterol sulfate.

In animal tissues, cholesterol (cholest-5-en-3β-ol) is the most abundant member of a family of polycyclic compounds known as sterols. Cholesterol biosynthesis involves numerous enzymes either cytosolic or endoplasmic reticulum-resident. The 3β-hydroxysterol Δ14-reductase (C14SR, EC 1.3.1.70), encoded by the Tm7sf2 gene, residing in the endoplasmic reticulum and recently found in the nucleous^1^, reduces the C14–C15 of unsaturated sterol intermediates^2^ in cholesterol biosynthesis. In order to define the *in vivo* functions of this protein, Tm7sf2-null mice were generated; these mice develop normally, are fertile and show no obvious abnormalities^3^. However, following tunicamycin treatment, Tm7sf2-null mice fail to increase hepatic cholesterol levels^4^, indicating that is essential for cholesterol biosynthesis, at least under stress conditions.

Cholesterol levels are of particular importance to the physiology and pathophysiology of the skin. For example, maintenance of cholesterol homeostasis in keratinocytes/epidermis is required to form lamellar bodies^5^,^6^. These fuse together in the stratum corneum to generate a continuous layer of lipids to form an impermeable barrier^7^,^8^. Disruption of this barrier up-regulates cholesterol synthesis^9^, along with levels of the receptors responsible for cholesterol uptake such as the low density lipoprotein receptor (Ldlr)^10^. On the other hand, inhibition of cholesterol synthesis perturbs barrier function^11^. Indeed deficiency in cholesterol synthesis largely accounts for the barrier abnormality in aged epidermis^12^,^13^. Beyond the barrier function of the epidermis, cholesterol synthesis is believed to be protective against a range of inflammatory and chemical insults to the skin^14^. Finally, cholesterol is the precursor of an important bioregulatory molecule in keratinocytes, cholesterol sulfate (CS), which regulates corneocyte desquamation and cohesion^15^ and keratinocyte differentiation^16^.

Given these important structural and protective roles of cholesterol in the skin, we used the tumour-promoting phorbol ester, which has been shown to disrupt sterol metabolism of mouse skin^17^, to define the role of Tm7sf2 in skin cholesterol homeostasis and susceptibility to tumour growth.

## Results

### Tm7sf2 gene drives cholesterol accumulation after TPA exposure

**Figure 1:**
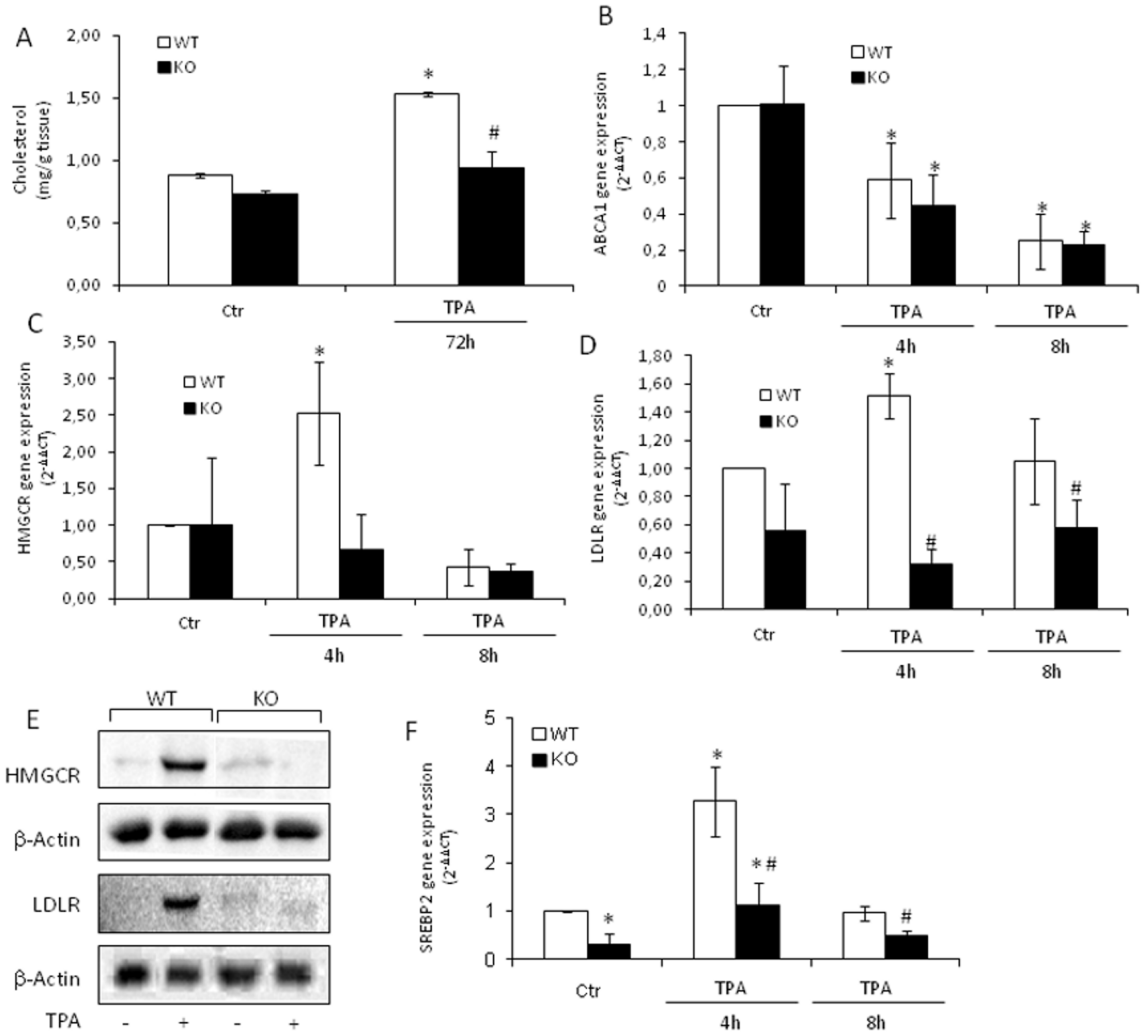
Tm7sf2 gene drives cholesterol accumulation after TPA exposure. WT and Tm7sf2 KO mice were subjected to a single skin topical application of TPA and sacrificed at the indicated time. The skin was removed and used to determine: (A) cholesterol levels by TLC analysis (n = 3); expression of (B) Abca1, (C) Hmgcr, (D) Ldlr, (F) SREB-2 by Real Time RT- PCR analyses. Expression of each gene was normalized to Gapdh and reported as 2^−ΔΔCt^. Relative mRNA level of WT untreated mice skin was assumed as 1. Results are given as mean ± S.D. (n = 4), *p < 0.05 vs. untreated WT, # p < 0.05 vs. the respective WT. (E) Whole skin lysates (pooled samples from n = 5 mice) were analyzed by western blotting with the indicated antibodies. Anti β-actin was used as loading control.

Cholesterol and/or its derivatives may play a direct role in epidermal homeostasis and TPA is known to change the metabolism of sterols in mouse skin^17^. To analyse the role of Tm7sf2 gene in cholesterol epidermal homeostasis we topically treated Tm7sf2^+/+^ and Tm7sf2^−/−^ mice with 20 nmol of TPA and determined cholesterol levels at 72 h. We found that the skin of Tm7sf2^+/+^ and Tm7sf2^−/−^ mice contained comparable basal amounts of cholesterol (0.88 ± 0.02 vs. 0.73 ± 0.01 mg g of tissue^−1^) and that TPA increased cholesterol levels only in the skin of Tm7sf2^+/+^ mice (Fig. 1A). It is to note that treatment with acetone did not affect skin cholesterol levels (Fig. S1 A and B).

Cholesterol accumulation may be due to: (i) increased *de novo* synthesis via up-regulation of 3-hydroxy-3-methylglutaryl-CoA reductase (Hmgcr), (ii) increased uptake of circulating cholesterol via the low-density lipoprotein receptor (Ldlr), and (iii) decreased cellular cholesterol efflux via the ATP-binding cassette transporter (Abca1). We found that TPA induced a decrease in Abca1 expression in both genotypes (Figure 1B), whereas it induced a significant increase in the mRNA levels of both Hmgcr and Ldlr only in the skin of Tm7sf2^+/+^ mice (Fig. 1C and D). Results were confirmed by Western blotting at 8 h TPA exposure (Fig. 1E). Consistent with these findings, sterol regulatory element binding protein 2 (SREBP-2), the key transcription factor that induces the expression of both Ldlr and Hmgcr^18^, was found markedly increased at 4 h TPA exposure in the skin of Tm7sf2^+/+^ mice (Fig. 1F). Interestingly, SREBP-2 gene expression was lower in Tm7sf2^-/-^ mice in basal conditions and was significantly up-regulated by TPA (Fig. 1 F), whereas its activation was induced by TPA independently of the genotype (Fig. S1C). Data indicate that the Tm7sf2 gene regulates cholesterol homeostasis by inducing *de novo* synthesis and up-take from circulation after TPA treatment.

### Tm7sf2 gene regulates cholesterol sulfate homeostasis

**Figure 2:**
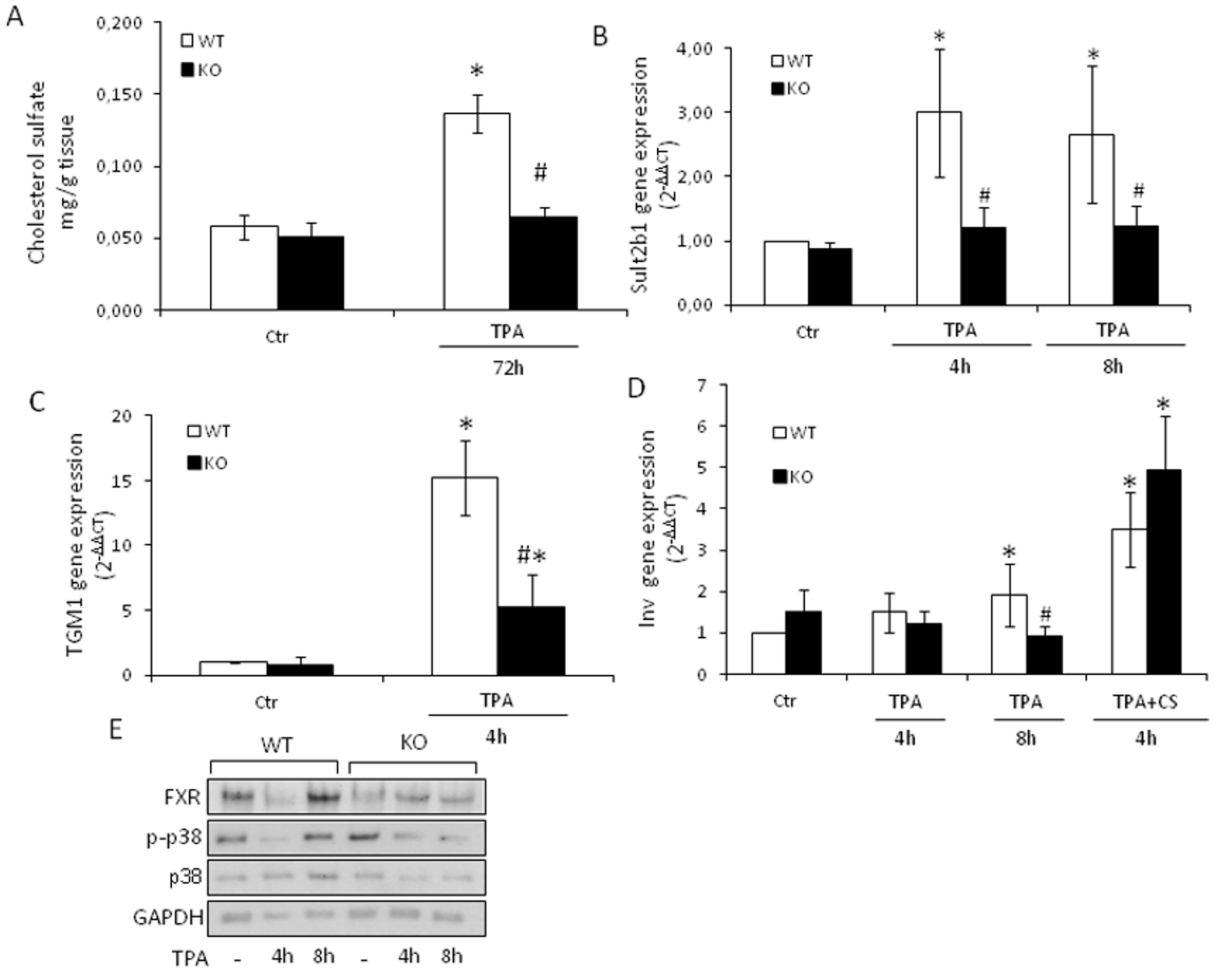
Tm7sf2 gene regulates cholesterol sulfate homeostasis. WT and Tm7sf2 KO mice were subjected to a single topical application of TPA and sacrificed at the indicated time. The skin was used to determine: (A) cholesterol sulfate levels by TLC analysis (n = 3); expression of (B) Sult2B1, (C) Tgm1, and (D) Inv by Real Time RT- PCR analyses. When cholesterol sulfate was used, mice of both genotypes were pre-treated with 820 μmol of cholesterol sulfate 10 min before the treatment with TPA for 4 h. Expression of each gene was normalized to Gapdh and reported as 2^−ΔΔCt^. Relative mRNA level of WT untreated mice skin was assumed as 1. Results are given as mean ± S.D. (n = 4), *p < 0.05 vs. untreated WT, # p < 0.05 vs. the respective WT. (E) Whole skin lysates (pooled samples from n = 5 mice) were analyzed by Western Blotting with the indicated antibodies. Anti β-actin was used as loading control.

Skin cholesterol is converted by cholesterol sulfotransferases (Sults) into cholesterol sulfate (CS) involved in keratinocyte differentiation and development of the epidermal barrier^19^. To assess whether Tm7sf2 can affect skin CS levels, we analyzed CS levels at 72 h TPA treatment (Fig. 2A). We found that the skin of Tm7sf2^+/+^ and Tm7sf2^−/−^ mice contained comparable basal amounts of CS (0.082 ± 0.02 vs. 0.068 ± 0.01 mg g of tissue^−1^) and that TPA induced a doubling in CS levels only in the Tm7sf2^+/+^ mouse skin (Fig. 2A).

Sult2B1 is the main isozyme of the Sult family active in sulfonating cholesterol^20^. Thus, to determine whether the loss of the Tm7sf2 gene can cause decreased expression of the Sult2B1 gene, we analyzed its expression up to 8 h TPA treatment and found that TPA induced a significant increase in Sult2B1 mRNA levels only in the Tm7sf2^+/+^ mouse skin (Fig. 2B). These data indicate that the Tm7sf2 gene regulates CS biosynthesis by increasing cholesterol levels and the expression of an enzyme involved in cholesterol sulfonation.

CS is implicated in the regulation of the early and late stages of keratinocyte differentiation^16^,^21^,^22^. We surmised that the markers of keratinocyte differentiation could be altered by the loss of the Tm7sf2 gene. Therefore, we treated mice of both genotypes with TPA and analysed the mRNA levels of transglutaminase 1 (Tgm1) and involucrin (Inv). We found that TPA exposure significantly increased Tgm1 and Inv mRNA levels only in Tm7sf2^+/+^ mouse skin (Fig. 2C and D) suggesting that the lack of Tm7sf2 alters the expression of markers of differentiation after TPA exposure.

To confirm the role of CS in Inv mRNA induction, we pre-treated mice of both genotypes with 820 μmol of CS 10 min prior to TPA treatment (Fig. 2D). We found that exogenous CS, while not affecting the TPA-induced HMGCR expression (Fig. S2), increased the Inv mRNA expression independently of the genotype, confirming the pivotal role for Tm7sf2 in keratinocyte differentiation.

The nuclear receptor Farnesoid-X-receptor (FXR) and p38 MAP kinase are deeply involved in skin development and functionality, the former by accelerating the formation of a mature stratum corneum^23^, the latter by up-regulating Inv expression^24^. Thus, we analysed by western blotting the activation of p38 and the expression of FXR (Fig. 2E) and found that TPA failed to induce their activation/expression in Tm7sf2^-/-^ mouse skin, indicating that Tm7sf2 regulates multiple signalling pathways responsible for epidermal differentiation.

### Tm7sf2 controls papilloma incidence and multiplicity

**Figure 3:**
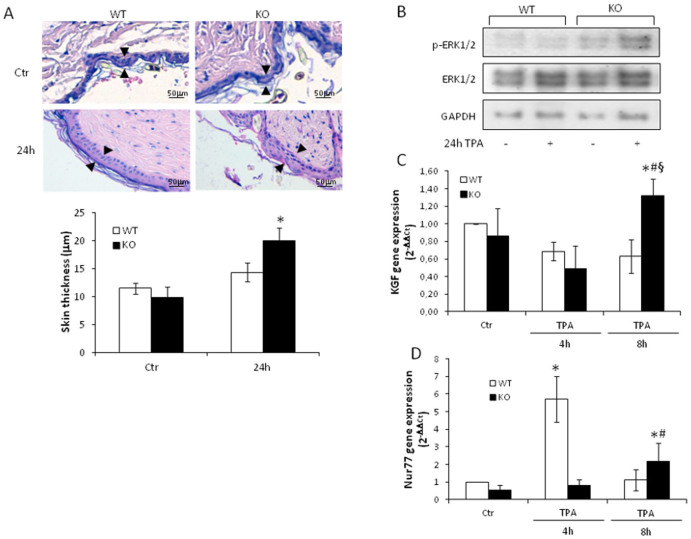
Tm7sf2 controls TPA-induced epidermal hyper-proliferation. WT and Tm7sf2 KO were subjected to a single skin topical application of TPA and (A) skin architecture and epidermal thickness were analysed with H&E staining (magnification 200×), arrows indicate epidermal thickness; quantification was performed on n = 4 skin samples, each measured in at least 3 different fields; (B) levels of phosphorylated ERK1/2 by western blotting (n = 5 pooled samples). *P < 0.05 vs control mice of the respective genotype. WT and Tm7sf2 KO mice were subjected to a single topical application of TPA and sacrificed at the indicated time. The skin was used to determine the expression of (C) KGF and (D) Nur77 by Real Time PCR analyses. Expression of each gene was normalized to Gapdh and reported as 2^−ΔΔCt^. Relative mRNA level of WT untreated mice skin was assumed as 1. Results are given as mean ± S.D. (n = 4), *p < 0.05 vs. untreated WT, # p < 0.05 vs. the respective WT.

Cholesterol levels have been linked to keratinocyte proliferation. Indeed, cholesterol depletion, acting through the EGFR, activates the extracellular signal-regulated kinases (ERK) 1/2^25^. To verify whether the loss of Tm7sf2 impairs keratinocyte proliferation, we treated the skin of Tm7sf2^+/+^ and Tm7sf2^−/−^ mice topically with a single dose of TPA and measured epidermal thickness at 24 h. We found that the TPA challenge induced a significant increase in epidermal thickness only in Tm7sf2^−/−^ mice (Fig. 3A). We verified by western blotting the activation of ERK1/2, a well-known driver of cell proliferation in this model^26^. We found increased phosphorylation of ERK1/2 only in the skin of Tm7sf2^−/−^ mice (Fig. 3B). Keratinocyte growth factor (KGF) via ERK 1/2 activation is responsible for epidermal cell proliferation^27^. Consistent with our results, we found that TPA exposure caused an increase in KGF expression in the skin of Tm7sf2^−/−^ mice at 8 h TPA exposure (Fig 3C). Results indicate that Tm7sf2 gene is involved in the control of skin proliferation after TPA challenge. To better elucidate the role of the Tm7sf2 gene in skin papilloma formation after TPA treatment, we analyzed the expression of the orphan nuclear receptor Nur77, linked to resistance to neoplastic transformation in the skin^28^. We found that a 4 h TPA exposure caused a marked increase in Nur77 expression only in the skin of Tm7sf2^+/+^ mice (Fig. 3D).

**Figure 4:**
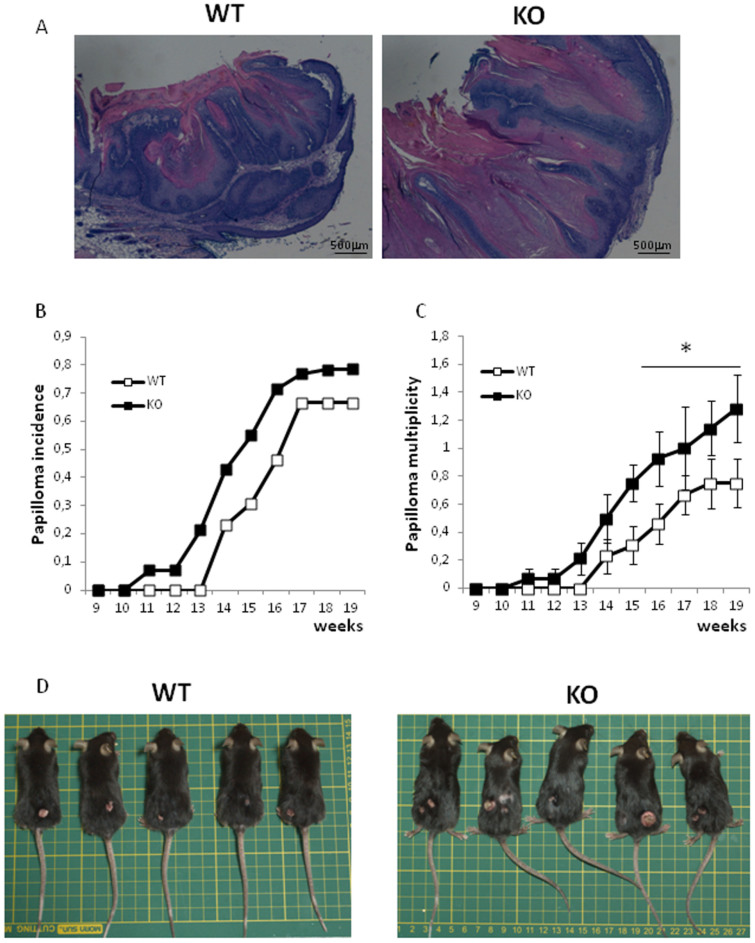
The lack of Tm7sf2 increases papilloma incidence and multiplicity. Tm7sf2 WT (n = 15) and KO (n = 15) mice were topically treated with a single dose of DMBA followed by bi-weekly applications of TPA for 19 weeks. (A) H&E staining of 4 μm sections from papillomas of Tm7sf2 WT and KO mice. (B) Papilloma incidence and (C) papilloma multiplicity at the indicated times after DMBA treatment. (D) Representative images of Tm7sf2 WT and KO papilloma-bearing mice (magnification 40×). *p < 0.05 vs control WT mice.

By mimicking the multistage nature of human cancer development^29^, the DMBA/TPA–induced two-stage skin carcinogenesis serves as a well-characterized *in vivo* mouse model for epithelial neoplasia. To assess the role of Tm7sf2 in skin tumourigenesis, we treated mice of both genotypes with DMBA/TPA. We observed that both genotypes developed papillomas with similar histological features (Fig. 4A). The first detectable tumour (>2 mm) appeared after 9 weeks of treatment in the null genotype and after 11 weeks in Tm7sf2^+/+^ mice. By week 15, 30% of Tm7sf2^+/+^ and 55% of Tm7sf2^−/−^ mice showed papillomas (Fig 4B). Even tumour multiplicity was higher in Tm7sf2^−/−^ animals and this difference reached statistical significance after 15 weeks of treatment (Fig. 4C). By week 19, papillomas developed in Tm7sf2^−/−^ mice were greater in size (17 ± 10 mm^2^ in WT vs 43.2 ± 14 mm^2^ in KO mice p = 0.039) (Fig. 4D). These results demonstrate that loss of Tm7sf2 markedly increases size, incidence and multiplicity of skin papillomas.

## Discussion

In the present study, we found that the loss of the Tm7sf2 gene markedly increases size, incidence and multiplicity of skin papillomas by altering skin cholesterol levels in a mouse model of skin carcinogenesis.

Consistent with previous reports^2^, Tm7sf2-null mice displayed normal epidermal morphology, indicating that Tm7sf2 is not essential for epidermal homeostasis under basal conditions. This could be explained by the possible functional compensatory redundancy of the lamin B receptor (LBR)^2^,^30^. However our data demonstrate unique roles for Tm7sf2 in the resistance to tumour development and in the control of cholesterol biosynthesis in the skin that follows TPA challenge; the up-regulation of the cholesterol biosynthetic machinery noted in wild type mice after TPA treatment was lost in the Tm7sf2 knock out animals while tumours in the knock out animals were more numerous, larger in size and developed more rapidly. The association between tumour development and loss of Tm7sf2 expression is consistent with published data showing reduced Tm7sf2 expression in both cancer cell lines and in primary adrenocortical tumour tissue as compared to the respective normal tissues^2^,^31^,^32^.

While the mechanism by which the loss of Tm7sf2 expression promotes tumour development in the skin remains to be definitively elucidated our data offer some tantalising clues. Firstly, proliferative signals and signalling pathways, important features of tumour development^33^,^34^, appear to be exaggerated in the absence of Tm7sf2. Thus, the TPA-induced production of KGF, which can induce skin hyper-proliferation^35^ and activation of the ERK pathway, through which it promotes proliferation and DNA synthesis^27^, are both augmented in Tm7sf2 null mice. Activation of ERK1/2 can also be caused by low cell cholesterol levels^25^,^36^. Secondly, other TPA-inducible pathways associated with resistance to neoplastic transformation, such as the orphan nuclear receptor Nur77^28^, are lost in the Tm7sf2 KO animals. While the target genes of this transcription factor in the skin are currently unknown, our data connect Tm7sf2 to both anti-proliferative and tumour resistance responses.

Cholesterol biosynthesis is essential for skin homeostasis and protection against toxic topical insults as evidenced by the abnormal cutaneous phenotypes associated with the loss of cholesterol biosynthetic enzymes^37^,^38^,^39^,^40^,^41^,^42^. Our data demonstrate a requirement for Tm7sf2 in a host of protective skin responses to TPA challenge. TPA-induced increases in skin cholesterol levels, elements of the cholesterol biosynthetic machinery (Hmgcr) and cholesterol uptake (Ldlr), as well as increased expression of the transcription factor (Srebp-2) responsible for driving expression of these genes^18^ detected in wild type animals, were all lost in Tm7sf2^−/−^ mice. Interestingly, TPA-induced SREBP-2 activation was independent of the genotype. Nevertheless, it has been demonstrated that Nur77 regulates cholesterol levels through the suppression of LDLR and HMGCR expression^43^, suggesting a pivotal role for this orphan receptor in the regulation of cholesterol metabolism.

At the same time, the activation of pathways associated with the returning cholesterol biosynthesis to basal levels 8 h after TPA challenge, such as the p38 MAPK pathway and the nuclear receptor farnesoid-X-receptor (FXR)^44^, are abolished in the Tm7sf2*^-/-^* mice. This presumably reflects the failure in these animals to initiate a complex program of gene expression designed to produce a pulse of cholesterol biosynthesis in response to TPA challenge. Notably the loss of these responses in other systems (e.g. hepatocellular carcinoma) is associated with a more aggressive proliferation^45^,^46^ consistent with the enhanced papilloma development observed in our skin model. Interestingly a second, related nuclear receptor, liver X receptor (LXR) which controls expression of the cholesterol exporter protein, Abca1, appeared unaffected by loss of Tm7sf2 because Abca1 expression was down regulated equally in both genotypes in response to TPA-challenge. This suggests that at least some TPA-mediated responses are independent of Tm7sf2.

The above effects relate to the importance of cholesterol in controlling skin physiology but its derivative, cholesterol sulfate, also functions as a key controller of epidermal differentiation. CS is synthesized from cholesterol by cholesterol sulfotransferase (Sult), which, in turn, is up-regulated by inducers of epidermal differentiation such as calcium^19^,^47^. We showed that the TPA-induced increases in CS levels and in the expression of Sult observed in wild type mice was lost in Tm7sf2 null mice. This finding may relate to the observed lack of increase of FXR in the null genotype since FXR small interfering RNA (siRNA) significantly reduces Sult2a1 promoter activity^48^. Thus, Tm7sf2 appears to control the level of CS by affecting both the level of cholesterol and its subsequent sulfonation by Sult.

CS drives differentiation of keratinocytes by activating protein kinase C leading to activation of the transcription factor activator protein-1 (AP1). This drives expression of several markers of differentiation such as Tgm1 and Inv^21^,^22^,^49^,^50^. In our experiments, we documented the expected increase in Tgm1 and Inv expression only in the skin of Tm7sf2^+/+^ mice. Notably, in the case of Inv, the deficit was rescued by the addition of exogenous CS suggesting that the observed reduction was a direct result of the lack of CS in Tm7sf2^−/−^ mouse skin. As expected, CS addition did not result in an increased expression of the up-stream gene Hmgcr. Since the expression of Inv is controlled by the p38 pathway^24^, this observation may relate to the failure of TPA to activate the p38 pathway in the Tm7sf2^−/−^ mice discussed above.

In conclusion, the data presented here strongly implicate Tm7sf2 in driving a host of protective responses after topical TPA challenge, centred on increased cholesterol and CS levels.

## Methods

### Materials

All the reagents, unless otherwise stated, were from Sigma Aldrich (St. Luis, MO). Cell culture reagents were from Life Technologies (GibcoBRL, Gaithersburg, MD).

### Animals

Tm7sf2^+/+^ and Tm7sf2^−/−^ mice, on a C57BL/6 background^3^ (>20 backcross), were housed at the Laboratory Animal Research Centre of Perugia University. The animals were maintained at a constant temperature of 22°C, 12 h light/dark cycle, and fed ad libitum.

### Ethics statement

All experimental procedures were carried out in accordance with European Directives, approved by the Institutional Animal Care and Use Committee of Perugia University (106/2012). Efforts were made to minimise animal stress/discomfort.

### TPA-induced inflammation in mouse skin

The shaved dorsal regions of female Tm7sf2^+/+^ and Tm7sf2^−/−^ mice (6–8 weeks of age) were treated topically with 20 nmol of TPA (12-o-tetradecanoylphorbol-13-acetate) in 100 μL of acetone or with acetone alone. Mice were sacrificed by CO_2_ asphyxiation 4, 8, 24 or 72 h after TPA treatment. The dorsal skin was excised, and the fat from the whole skin was removed on ice. The fat-free skin tissues were then frozen immediately in liquid nitrogen or fixed in formalin. When cholesterol sulfate was used, mice of both genotypes were pre-treated with 820 μmol of cholesterol sulfate 10 min before TPA treatment.

### Two-stage skin carcinogenesis protocol

The shaved dorsal regions of female Tm7sf2^+/+^ and Tm7sf2^−/−^ mice (6–8 weeks of age) were treated topically with a single dose of 0.2 mmol DMBA (7,12-dimethylbenz[α]anthracene) dissolved in 100 μL of acetone. One week later the mice were treated topically with 20 nmol of TPA in 100 μL of acetone (n = 15) or acetone alone (n = 5) twice weekly for 19 weeks. The incidence and number of tumours that were at least 2 mm in diameter were monitored and counted weekly. The results were expressed as the percentage of tumour-bearing mice (tumour incidence) and the average number of tumours per mouse (tumour multiplicity).

### Western Blotting

Skin tissue (20% w/v) was lysed in a boiling Laemmli sample buffer, resolved on SDS-polyacrylamide gels and transferred to nitrocellulose membranes. Membranes were probed with anti Hmgcr, anti Ldlr (Abcam, Cambridge, UK), anti phosphor-p38 (Tyr180/Tyr182) (Cell signaling technology, Danvers, MA), anti FXR, anti SREBP-2 (CRL-2545) (ATCC), anti Gapdh and anti β-actin (Santa Cruz Biotechnology, Santa Cruz, CA) which were detected using HRP-based chemiluminescence (ECL, Pierce Biotechnology, Rockford, IL). Each sample was prepared identically from five individual animals and, within each group, results were highly reproducible.

### Real Time RT-PCR

Total RNA was isolated with TRI Reagent according to the manufacturer's instructions and cDNA was synthesised using iScript cDNA synthesis kit (Bio-Rad Lab, Hercules, CA). Real time PCR was performed using the iCycler iQ detection system (Bio-Rad Lab, Hercules, CA) and SYBR Green chemistry. Primers are listed in Table 1. SYBR Green RT-PCR amplifications were carried out in a 96-well plate in a 25 μL reaction volume that contained 12,5 μL of SYBR® Green JumpStart™ *Taq* ReadyMix™, 400 nmol L^−1^ forward and reverse primers, and 5 to 40 ng of cDNA. In each assay, no-template controls were included and each sample was run in triplicate. Mean of C_t_ values of the samples was compared to the untreated control sample and Gapdh used as internal control. The n-fold differential ratio was expressed as 2^−ΔΔCt^.

### Histochemistry

Paraffin-embedded sections from mouse skin were collected for immunohistochemical (IHC) analysis. Briefly, tissue samples were fixed in 10% buffered formalin and embedded in paraffin. The 4 μm tissue sections were stained with hematoxylin & eosin.

### Determination of cholesterol and cholesterol sulfate by TLC

Skin explants (50 mg) were incubated overnight at 55°C in 0.2 mL of lysis buffer (0.1 mol L^−1^ Tris-HCl, pH 8.0, 12.5 mmol L^−1^ EDTA, 0.2 mol L^−1^ NaCl, and 0.25 mg mL^−1^ proteinase K), then lipids were extracted by the Bligh and Dyer method in the presence of 1 mol L^−1^ KCl to allow full cholesterol sulfate recovery^51^. Cholesterol was analyzed by TLC as previously described^3^. CS was analyzed by TLC using diethyl ether/n-hexane/acetic acid (45:40:15) as developing mixture. Plates were stained with Cu-acetate/phosphoric acid^52^. Images were acquired using the VersaDoc Imaging System and signals were quantified using Quantity One software (Bio-Rad, Milan, Italy) and pure cholesterol and cholesterol sulfate standards for reference.

### Statistical Analysis

The results were expressed as the mean ± SD and analyzed via analysis of variance. Differences between the treatment groups were evaluated via Student's t-test, except the differences in tumour incidence and multiplicity that were analyzed via a two-sample test for binomial proportions. Differences were considered significant at p < 0.05.

## Author Contributions

I.B., A.M., R.R. and M.J.P. designed the experiments, interpreted the results and wrote the paper. A.S. and R.dS. performed histochemical analysis. L.G. and I.B. performed the experiments. All authors reviewed the manuscript.

## Supplementary Material

Supplementary Information Supplementary Material

## Figures and Tables

**Table 1:** List of primers

Gene name	Gene symbol	Primer sequence (F: Forward; R: Reverse)
ATP-binding cassette transporter 1	Abca1	F: CCAGACGGAGCCGGAAGGGT
		R: GTGCCCATGTCCTCGGGAGC
Glycerhaldeyde 3-phosphate dehydrogenase	Gapdh	F:GCCAAATTCAACGGCACAGT
		R:AGATGGTGATGGGCTTCCC
HMG-CoA reductase	Hmgcr	F: TGCCTGGATGGGAAGGAGTA
		R: GCCTCGAGTCATCCCATCTG
Involucrin	Inv	F: CCCTCCTGTGAGTTTGTTTGG
		R: TGAGAGGTCCCTGAACCACA
Keratinocyte growth factor	KGF	F: ACGGCTACGAGTGTGAACTG
		R: GGGTCCCTTTCACTTTGCCT
LDL Receptor	Ldlr	F: GGGAACATTTCGGGGTCTGT
		R: AGTCTTCTGCTGCAACTCCG
Nuclear receptor subfamily 4 group A member 1	Nr4a1 (Nur77)	F: TGTGCTAGAAGGACTGCGGA
		R: ATGGTAGGCTTGCCGAACTC
Sterol regulatory element-binding protein 2	Srebp-2	F: CAAGCACACTGATTGAGAT
		R:TGGGCACGATTTAAGAAGTA
Cytosolic Sulfotransferase 2B1	Sult2B1	F: GCCCAGAGTGAGGCTTTTGA
		R: CCTCTTCAGACGTGTCCCAG
Tranglutaminase 1	Tgm1	F: CTGTTGGTCCCGTCCCAAAC
		R: GGACCTTCCATTGTGCTGGAG
