# Supplementary material for: The loss of Tm7sf gene accelerates skin papilloma formation in mice
Source: Sci Rep. 2015 Mar 25;5:9471. doi: 10.1038/srep09471 (PMC4372794; doi:10.1038/srep09471)
Supplement: Supplementary Information — Supplementary Material [file srep09471-s1.pdf]

The loss of Tm7sf gene accelerates skin papilloma formation in mice

I. Bellezza *, L. Gatticchi, R. del Sordo, M.J. Peirce, A. Sidoni, R. Roberti, A. Minelli

Supplementary Materials and Methods

Oil red staining

Skin samples were embedded in OTC reagent (Tissue-Tek; Sakura Finetek, Hatfield, PA), frozen and sectioned at 8 µm in cryostat. Cryosections were fixed in formaldehyde vapor at 50°C, incubated in 0.5% oil red O in isopropyl alcohol, and counterstained with hematoxylin.

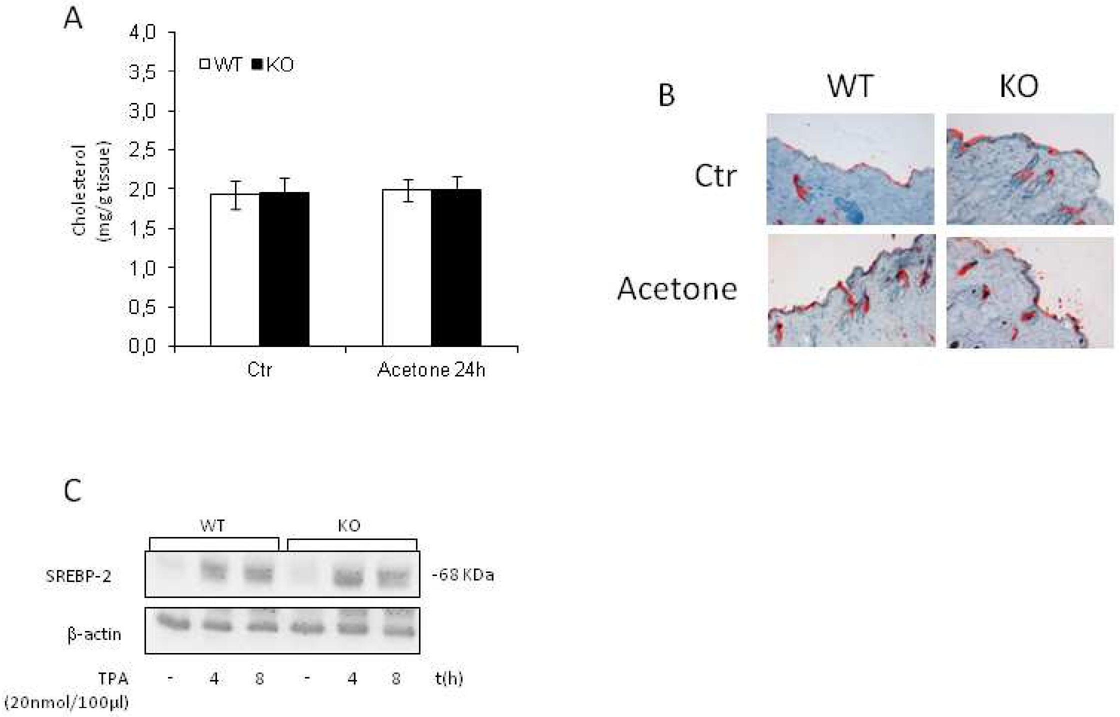

Figure S1. Related to Fig 1.

WT and Tm7sf2 KO mice were subjected to a single skin topical application of acetone and sacrificed after 24h. The skin was removed and used to determine: A) cholesterol levels by TLC analysis; B) Neutral lipid distribution by Oil Red O staining. Magnification 20x. WT and Tm7sf2 KO mice were subjected to a single skin topical application of TPA and sacrificed at the indicated time.

C) Whole skin lysates (pooled samples from n=5 mice) were analyzed by Western Blotting with the indicated antibodies. Anti β-actin was used as loading control.

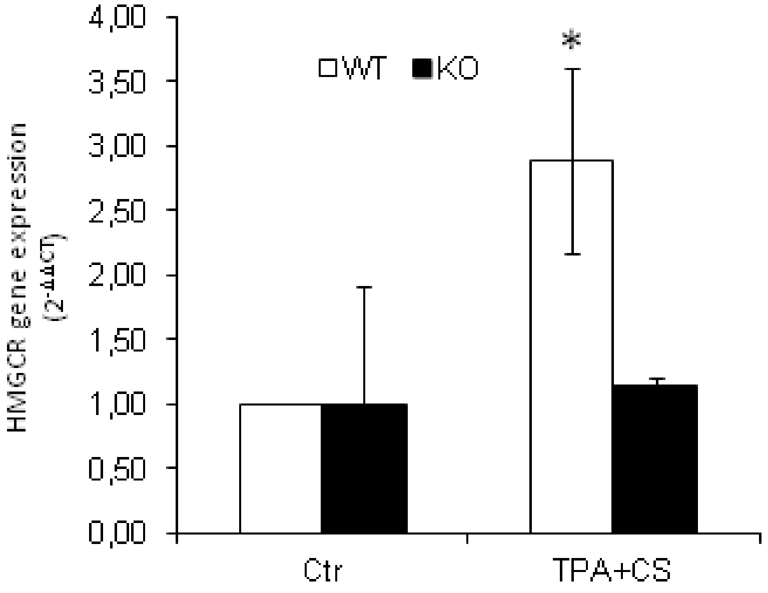

Figure S2. Related to Fig 2.

WT and Tm7sf2 KO mice were pre-treated with 820µmol of cholesterol sulfate 10min before the treatment with TPA for 4h. The skin was used to determine the expression of HMGCR by Real Time RT- PCR analyses. Expression of the gene was normalized to Gapdh and reported as $2^{-\Delta\Delta}$Ct. Relative mRNA level of WT untreated mice skin was assumed as 1. Results are given as mean ± S.D. (n=4), *p<0.05 vs. untreated WT.
